# TRPM7 overexpression enhances the cancer stem cell-like and metastatic phenotypes of lung cancer through modulation of the Hsp90α/uPA/MMP2 signaling pathway

**DOI:** 10.1186/s12885-018-5050-x

**Published:** 2018-11-26

**Authors:** Kai Liu, Shao-Hua Xu, Zhao Chen, Qing-Xin Zeng, Zhi-Jun Li, Zhou-Miao Chen

**Affiliations:** Department of Thoracic Surgery, Sir Run Run Shaw Hospital, School of Medicine, Zhejiang University, 3 East Qing Chun Road, Zhejiang Province, 310016 Hangzhou China

**Keywords:** Lung Cancer, TRPM7., Waixenicin A., cancer stem cells.

## Abstract

**Background:**

Waixenicin A, a bioactive extract of soft coral *Sarcothelia edmondsoni*, has been shown to be anti-neoplastic. However, its mechanisms of action remain unclear. Cancer stem cells (CSCs) and associated stemness factors are implicated in lung cancer. Here, we investigated the role of Waixenicin A on CSCs-like and metastatic lung cancer cells.

**Methods:**

We demonstrated and compared TRPM7 expression in the non-tumor lung tissues or bronchial epithelial 16-HBE cell line. TRPM7 was aberrantly expressed in the cancer tissues and SPCA-1, NCI-H520, SK-MES-1, A549 and 95D cell lines.

**Results:**

Increased *TRPM7* expression was associated with enhanced *SOX2, KLF4*, and *CD133*, *Hsp90α, uPA,* and *MMP2* expression in lung cancer cells. TRPM7-silencing inhibited epithelial-to-mesenchymal transition (EMT), suppressed stemness markers and phenotypes, concomitantly suppressed Hsp90α/uPA/MMP2 axis. Coincidently, Waixenicin A treatment downregulated TRPM7 and oncogenic markers; Waixenicin A also attenuated the ability of lung cancer cells to form tumorspheres, in vitro. In validation, our clinicopathological analyses showed that a higher TRPM7 expression was positively correlated with the larger tumor size (*p* = 0.007), positive lymph node metastasis (*p* = 0.005) and disease grade (*p* = 0.003).

**Conclusions:**

Through its ability to inhibit Hsp90α/uPA/MMP2 signaling and suppress TRPM7 expression, we showed that Waixenicin A is a potential anticancer therapeutic agent for treating malignant lung cancer.

**Electronic supplementary material:**

The online version of this article (10.1186/s12885-018-5050-x) contains supplementary material, which is available to authorized users.

## Research highlights

1. TRPM7 overexpression enhances the cancer stem cell-like and metastatic phenotypes
2. TRPM7 modulation of the Hsp90α/uPA/MMP2 signaling pathway
3. Waixenicin A is a potential anticancer therapeutic agent with TRPM7 modulation efficacy

## Background

Lung cancer, with an estimated incidence of 1.8 million cases and constituting approximately 13% of all new cancer diagnoses, is the most common malignancy and the leading cause of cancer-related deaths, globally [1]. With a high fatality of 0.87 as estimated by the overall mortality to incidence ratio, and a relatively low 5-year survival rate of 17.8%, lung cancer has one of the poorest prognoses among all cancers [2]. With less than 40% of lung cancer patients being eligible for surgical resection due to late/ advanced stage presentation, prognosis remains poor, as many patients do not survive regardless of chemo- and/or radio-therapy, and early metastases with high recurrence rate continue to plague therapeutic efforts [3], thus, necessitating the discovery or development of more effective therapeutic strategies to limit chemoresistance, inhibit formation of micrometastases and prevent disease recurrence.

In the last decade, the role of cancer stem cells (CSCs) in the causation of chemotherapy failure and their therapeutic implication as potential targets for prevention of malignancies, including lung cancer has garnered increased interest. The lung CSCs model suggest that the malignant phenotype of lung cancer cells is sustained by a sub-population of cells with enhanced capacity for self-renewal, differentiation and intrinsic resistance to contemporary chemotherapy and radiation. These CSCs are increasingly implicated in disease recurrence after definitive therapy and functionally associated with minimal residual disease [4, 5], especially as it has been suggested that conventional chemotherapeutic strategies mainly target proliferating cells, while the CSCs remain untouched by manipulating the multidrug resistant mechanisms, thereby facilitating the relapse of cancer [6]. The delivery or administration of drugs that target and eliminate CSCs may constitute a more efficient therapeutic strategy in the treatment of patients with recurrent or advanced stage lung cancer [7, 8]. For this reason, drugs that selectively target CSCs offer a greater promise for cancer therapy and prevention.

Evidence continue to accumulate that dietary phytochemicals or plant extracts possess cancer preventive and/or limiting potential, as demonstrated by their ability to eliminate transformed/malignant cells through various anti-cancer mechanisms, in vitro and in in vivo tumor xenograft models [9]; more so, some phytochemicals have demonstrated the ability to target CSCs by modulating genes and signaling pathways that facilitate the self-renewal capacity of CSCs and their associated drug resistance [10, 11].

Recent studies continue to outline the involvement of intracellular ion channels such as potassium or sodium channels in cancer development and progression, especially with their aberrant expression in malignancies and contribution in cellular proliferation, evasion of cell death, metabolic reprogramming, neovascularization, and enhanced motility of tumor cells [12, 13]. However, aside their possession of an ion channel and functional α-kinase domain, as well as their implication in Mg^2+^ homeostasis, the role of non-selective cation Transient Receptor Potential (TRP) channels in human lung cancer remains underexplored. The TRP superfamily currently consist of 28 mammalian members divided into 6 subgroups based on the homology of their sequence, namely ankyrin-like (TRPA), canonical (TRPC 1–7), polycystin (TRPP 1–5), mucolipin (TRPML 1–3), vanilloid (TRPV 1–6), and melastatin (TRPM 1–8) [14]. Unlike TRPM1 that plays the role of a tumor suppressor gene in melanoma, TRPM7 and TRPM8 are aberrantly expressed in pancreatic, gastric, breast and human head and neck cancers [15, 16]. Moreover, TRPM7 has been shown to regulate CSCs in glioma by modulating Notch and STAT3 signaling pathways [17, 18], however, it’s probable role in the modulation of lung cancer stem cells, induction of metastatic and drug-resistant phenotypes, its facilitation of poor prognosis, and likely targetability by therapeutic agents are unexplored in lung cancer patients and serve as the subject of our present study. Waixenicin A is a bioactive organic extract of the soft coral *Sarcothelia edmondsoni* (otherwise known as *Anthellia edmondsoni*), which has been shown to effectively inhibit TRPM7-mediated Mn^2+^ influx at concentrations as low as 30 μg/mL [16], modulate intestinal motility and regulate the pathophysiology of human gastric and breast adenocarcinoma cells [19]. Thus, in the present study, we investigated the probable role of TRPM7 in the modulation of lung cancer stem cells, induction of metastatic and drug-resistant phenotypes, and its facilitation of poor prognosis, as well as the inhibitory role of Waixenicin A on likely TRPM7-mediated CSCs-like and metastatic phenotypes of malignant lung cells.

## Methods

### Drugs and reagents

Waixenicin A was obtained from Huienna Chemical Export and Import Company (Tian Jin, China), Cisplatin (≥ 99.9% trace metal basis, 479,306 ALDRICH, Sigma-Aldrich, CA, USA) and Doxorubicin (≥ 98% HPLC, Sigma-Aldrich, CA, USA) were suspended in DMSO, prepared at a stock concentration of 1 mg/ml and stored at 20 °C.

### Cell lines and cell culture

Two NSCLC adenocarcinoma cell lines (SPCA-1 and A549) and two NSCLC squamous carcinomas cell lines (NCI-H520 and SK-MES-1) and one lung giant cell carcinoma cell line (95D) as well as SV40-immortalized human bronchial epithelial 16-HBE cell line were purchased from the Chinese Academy of Sciences Cell Bank and were cultured in Dulbecco’s Modified Eagle’s Medium (DMEM, Gibco, Carlsbad, CA, USA) supplemented with 10% fetal bovine serum (FBS), 2 mM L-glutamine 100 U/ml penicillin and 100 μg/ml streptomycin (Thermo Fisher Scientific, Inc. Waltham, Ma, USA) in a 5% CO_2_ humidified atmosphere at 37 °C. Cultured cells were passaged ≥98% confluence or media changed every 72 h.

### Sulforhodamine B colorimetric cell viability assay

1 × 10^5^ cells/mL A549 or 95D cells were plated per well containing 200 μl medium in 96-well culture plates overnight, then the cells were treated with different concentrations of Waixenicin A for 12–72 h, similarly, cells were treated with 0–20 μM Cisplatin or Doxorubicin for 48 h. Cell viability was examined at specified concentrations of named drugs. After exposure to treatment, cell viability was estimated using the sulforhodamine B assay as previously described [20]. Each experiment was performed at least three times in triplicate, and results are expressed as the mean ± SD.

### Western blot analysis

25 μg of total cell lysates were subjected to a 10% polyacrylamide sodium dodecyl sulfate - polyacrylamide gel electrophoresis (SDS-PAGE) and blots transferred onto polyvinylidene difluoride (PVDF) membranes. The membranes were then incubated with 5% non-fat milk in PBS with Tween-20 for 1 h to exclude non-specific binding before incubation overnight at 4 °C in specific primary antibodies against TRPM7, Vimentin, p21, Bak1, SOX2, KLF4, CD133, HSP90α, uPA, MMP2, Survivin, and STAT3 (Santa Cruz Biotechnology, CA, USA) followed by incubation in peroxidase - conjugated secondary antibody at room temperature for 1 h, washed with PBST three times, then the protein signals were observed using the UVP BioSpectrum system (Analytic Jena Company).

### Big data analysis

TRPM7 gene expression data in lung large-cell carcinoma (LCC), adenocarcinoma (ADC), squamous cell cancer (SCC) and normal lung tissue were accessed via the Gene Expression Omnibus (GEO) dataset browser using the series accession number GSE19188.

### Quantitative real-time PCR

The RNA expression levels in the treated and untreated control lung cancer cells were measured using a RT-PCR system. After extraction of total RNA using TRIzol reagent (Invitrogen, Thermo Fisher Scientific Inc., Carlsbad, CA, USA), 1 μg of the total RNA was reverse-transcribed using the QuantiTect Reverse Transcription Kit (Qiagen, Germantown, MD, USA). The amplified mRNA level of each specific stemness gene was normalized to that of GAPDH. All procedure was performed following manufacturers’ instruction.

### Small-interfering RNA transfection

The shRNA sequence targeting TRPM7 gene was F: 5’-AACTGGCACCTTTATATCATTAATT CAAGAGATTAATGATATAAAGGTGCCTTTTTTC-3′, R: 5’-GAAAAAAGGCACCTTTA TATCATTAATCTCTTGAATTAATGATATAAAGGTGCCAGTT-3′ were synthesized by GenePharma (Shanghai, China). shRNAs were transfected into lung cancer cells using Lipofectamine 2000 (Invitrogen), following the manufacturer’s instructions. Transfection efficiency was then assessed using quantitative real-time RT-PCR and Western blotting for TRPM7 expression. Cells with stably transfected shTRPM7 were cultured for further experiments.

### Tumor sphere formation assay

We generated tumorspheres by seeding 1 × 10^4^ control 95D cells or knockdown clones (shTRPM7 clones 1 and 2) per well in ultra-low adhesion 6-well plates, containing 2 mL warm StemXVivo serum-free tumorsphere media (CCM012, R&D Systems, Minneapolis, MN, USA) supplemented with 2 U/mL heparin (Sigma) and 0.5 μg/mL hydrocortisone (Sigma) following the manufacturer’s protocol, and cells incubated in 5% CO2 incubator, at 37 °C for 7–10 days. Tumor spheres were then observed under microscope and those larger than 50 μm counted.

### Colony formation assay

For anchorage-independent growth, 1 × 10^3^ A549 or 95D control cells or TRPM7 knockdown clones were suspended per ml in 2 ml of 0.3% agar with 1% N2 Supplement (Invitrogen), 2% B27 Supplement (Invitrogen), 20 ng/ml human platelet growth factor (Sigma-Aldrich), 100 ng/ml epidermal growth factor (Invitrogen) and 1% antibiotic-antimycotic (Invitrogen) overlaid into 6-well plates containing a 0.5% agar base. Formed colonies were stained with 2% crystal violet on day 21, then colonies ≥0.2 mm in diameter were observed and counted under microscope.

### Side-population identification assay

For identification of SP cells, 1 × 10^6^ 95D control cells or TRPM7-knockdown clones were incubated in 5 μg/mL Hoechst 33342 dye (Molecular Probes, Thermo Fisher Scientific) for 90 min with or without 50 μm verapamil. Cell samples were analyzed and sorted using a BD FACSAria™ III cell sorter (BD Biosciences) and SUMMIT software for data acquisition and analysis. Fluorescence emission was collected with a 405/30 nm and 670/40 nm band pass filter for Hoechst blue and Hoechst red, respectively. Dead cells were excluded using propidium iodide fluorescence at 670/30 nm.

### Transwell Matrigel invasion assay

Evaluation of the invasive capacity of A549 or 95D cells was performed using Transwell Filter (8 μm pore size, Corning Costar Corporation, Cambridge, MA) with Matrigel (BD Biosciences). Control cells or TRPM7-knockdown clones were plated in the upper chamber containing 200 μL serum-free culture medium, while the bottom chambers contained 500 μL of complete culture medium. After incubation for 24 h, the non-invaded cells in the upper chamber were carefully removed with cotton swap, and the invaded cells in the membrane of the lower chambers were stained with Giemsa stain. The stained cells were observed, photographed and counted under microscope in at least 5 randomly-selected fields.

### Transwell migration assay

For the cell migration assay, Transwell membranes (8 μm pore size, 6.5 mm diameter, Corning Costar Corporation, Cambridge, MA) were used. 5 × 10^3^ A549 or 95D cells were collected, washed, and re-suspended in serum-free medium in the upper chambers, while the lower chamber wells contained complete culture medium. After incubation for 24 h, the cells remaining on the upper membrane were carefully removed with cotton swap, while the migrated cells in the membrane of the lower chamber were fixed with 95% ethanol and stained with 0.1% crystal violet. 5 fields were randomly selected, and images captured under microscope at a magnification of × 200. Experiments were performed 3 times in triplicate.

### Immunofluorescence assay

Human lung cancer cells were fixed in 10% paraformaldehyde for 30 min, blocked with goat serum for 30 min, then incubated with anti-TRPM7 and anti-SOX2 antibodies (Santa Cruz Biotechnology) at a dilution of 1:100 for 1 h at 37 °C. Cells were washed 3 times with PBS, incubated with conjugated fluorescence isothiocyanate (FITC) for 1 h at 37 °C. DAPI was used for nuclear staining. The fluorescence intensity and cellular localization were evaluated under fluorescent microscope.

### Statistical analyses

SPSS v.18.0 for Windows software (SPSS Inc. Chicago, IL, USA) was used for statistical analysis. All data are expressed as mean + SEM of experiments performed independently at least twice in triplicate. One-way ANOVA and student’s t- test were used to determine the statistical differences between treatment groups. A *p* value < 0.05 was considered statistically significant.

## Results

### TRPM7 is aberrantly expressed in lung cancer tissue samples and cell lines

**Fig. 1:**
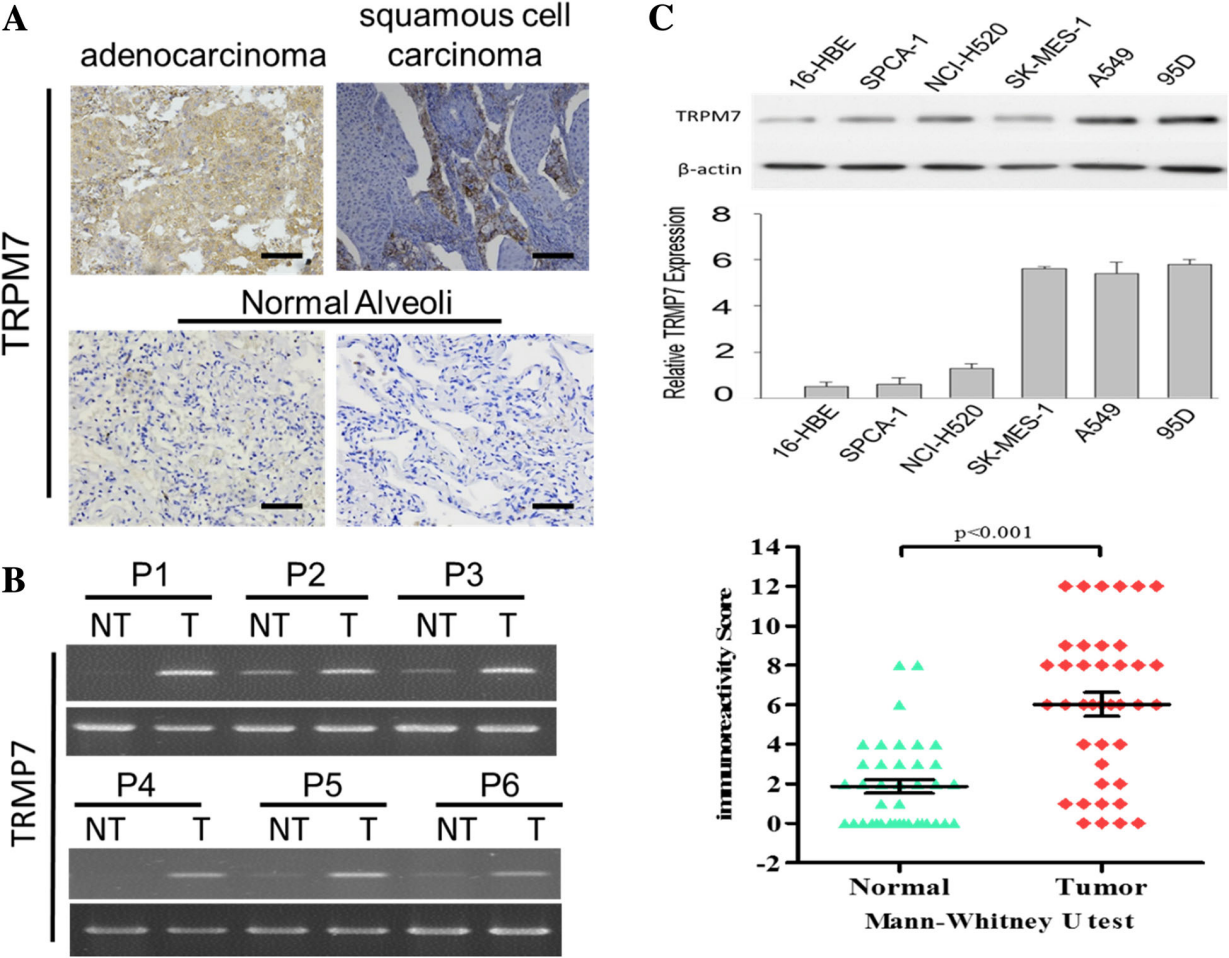
TRPM7 is aberrantly expressed in lung cancer tissue samples and cell lines. **a** Image showing that TRPM7 is strongly expressed in lung adenocarcinoma and squamous cell carcinoma, in comparison to weak expression in normal alveoli. **b** TRPM7 mRNA expression is upregulated in lung tumor tissues, compared to non-tumor tissues. **c** Compared to normal lung epithelial 16-HBE cell, TRPM7 protein expression level is upregulated in SPCA-1, NCI-H520, SK-MES-1, A549 and 95D lung cancer cell lines. β-actin served as loading control. *p < 0.05, **p < 0.01, ***p < 0.001

To understand the role of TRPM7 in lung cancer, we analyzed the differential expression profile of TRPM7 in paired lung adenocarcinoma or squamous cell lung carcinoma and adjacent normal alveoli tissue samples from our lung cancer cohort, using immunohistochemical (IHC) staining. Analysis of our data revealed that compared with the null or weak TRPM7 expression in normal alveoli samples, TRPM7 was strongly expressed in lung adenocarcinoma or squamous cell lung carcinoma (Fig. 1a). This IHC finding was corroborated by western blot analyses showing significantly enhanced TRPM7 protein expression level in lung tumor (T) compared to the adjacent non-tumor (NT) tissues (3.4-fold, *p* < 0.01) (Fig. 1b). Similarly, while TRPM7 protein expression was weak in the SV40-immortalized human bronchial epithelial 16-HBE cell line, TRPM7 was moderately expressed in SPCA-1 and NCI-H520, and strongly expressed in the SK-MES-1, A549 and 95D human lung cancer cell lines (Fig. 1c). These data showing aberrantly expressed TRPM7 in lung cancer tissue samples and cell lines are suggestive of the oncogenic role of TRPM7 in lung cancer cells.

### TRPM7 is an independent indicator of poor prognosis in lung cancer

**Fig. 2:**
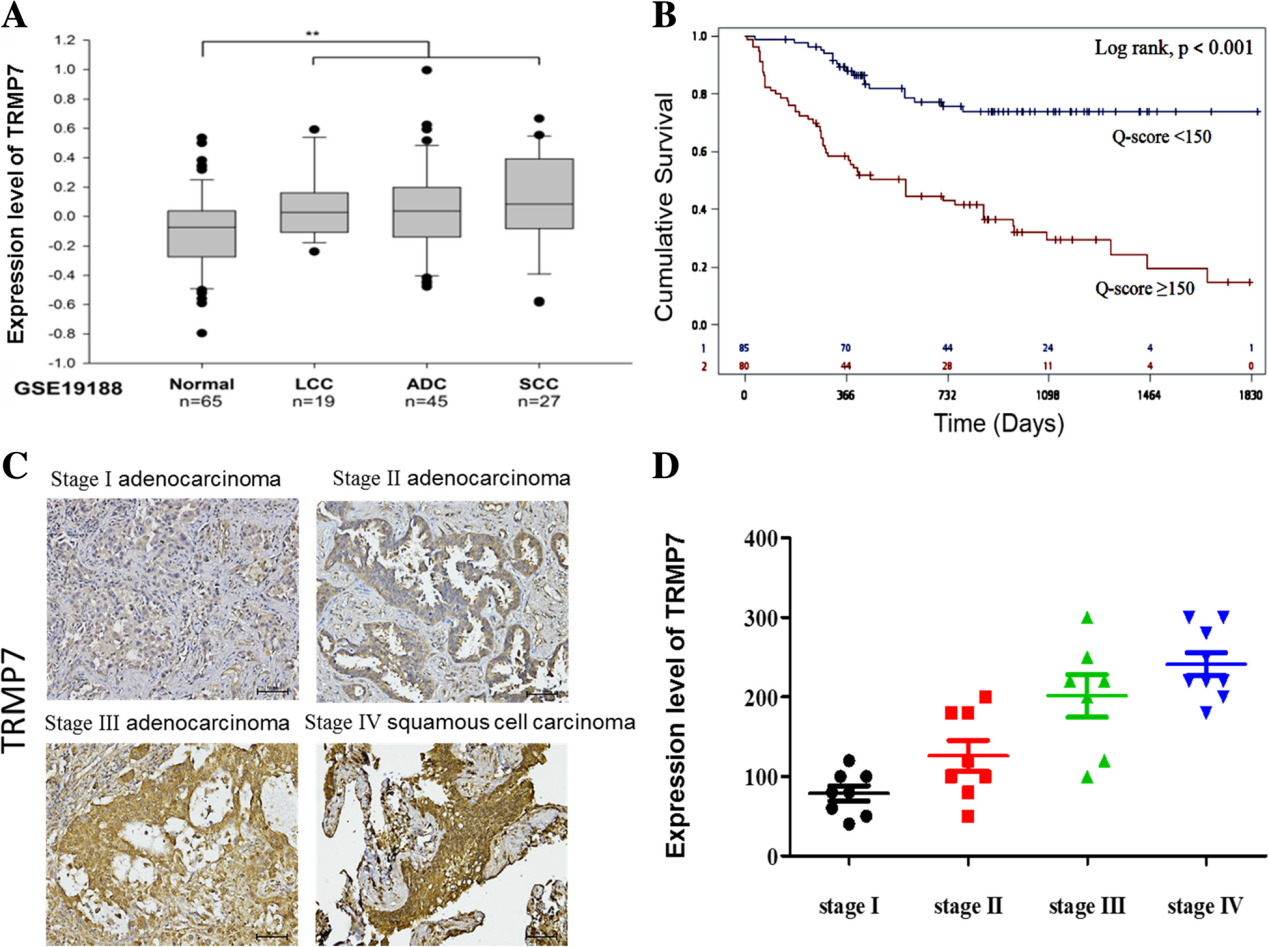
TRPM7 is an independent indicator of poor prognosis in lung cancer. **a** Relative mRNA expression of TRPM7 in lung large cell carcinoma, adenocarcinoma, squamous cell carcinoma and adjacent normal tissues in GSE19188 dataset, *n* = 156. **b** Kaplan-Meier analysis of TRPM7 gene expression in Sir Run Run Shaw hospital lung cancer cohort. Patients with low TRPM7 expression had longer cumulative survival than those with high TRPM7 expression. **c** Photo images and (**d**) graphical representation of the differential expression of TRPM7 in Stage I, II, III and IV lung cancers. * *p* < 0.05, ** *p* < 0.01, *** *p* < 0.001

For a functional characterization of TRPM7 in lung cancer, we accessed and analyzed TRPM7 gene expression profile in early stage NSCLC dataset with the series accession number GSE19188 and consisting of large-cell carcinoma (LCC, *n* = 19), adenocarcinoma (ADC, *n* = 45), squamous cell cancer (SCC, *n* = 27) and normal lung tissue (*n* = 65) were accessed via the Gene Expression Omnibus (GEO) browser. Consistent with earlier findings, we observed that relative to its expression in normal lung tissue, TRPM7 gene expression in the LCC, ADC and SCC samples was significantly elevated (*p* < 0.01) (Fig. 2a). Furthermore, TRPM7-specific survival analysis of our lung cancer cohort showed that patients with high expression of TRPM7 exhibited worse cumulative survival than those with low TRPM7 expression (*p* < 0.001), with a 46.0 and 59.1% difference in survival at third and fifth year, respectively (Fig. 2b and Additional file 1: Figure S1). We also demonstrated that TRPM7 expression positively correlated with tumor stage, as we observed a 1.9-fold, 2.4-fold, and 3.6-fold increase in the expression of TRPM7 in stage II, stage III and stage IV, when compared with its expression in stage I (Fig. 2c and d). These findings not only confirm the oncogenic role of TRPM7 in ling cancer but are indicative of the potential role of TRPM7 as an independent indicator of poor prognosis in lung cancer.

### Downregulation of TRPM7 expression suppresses the viability and metastatic phenotype of human lung cancer cells

**Fig. 3:**
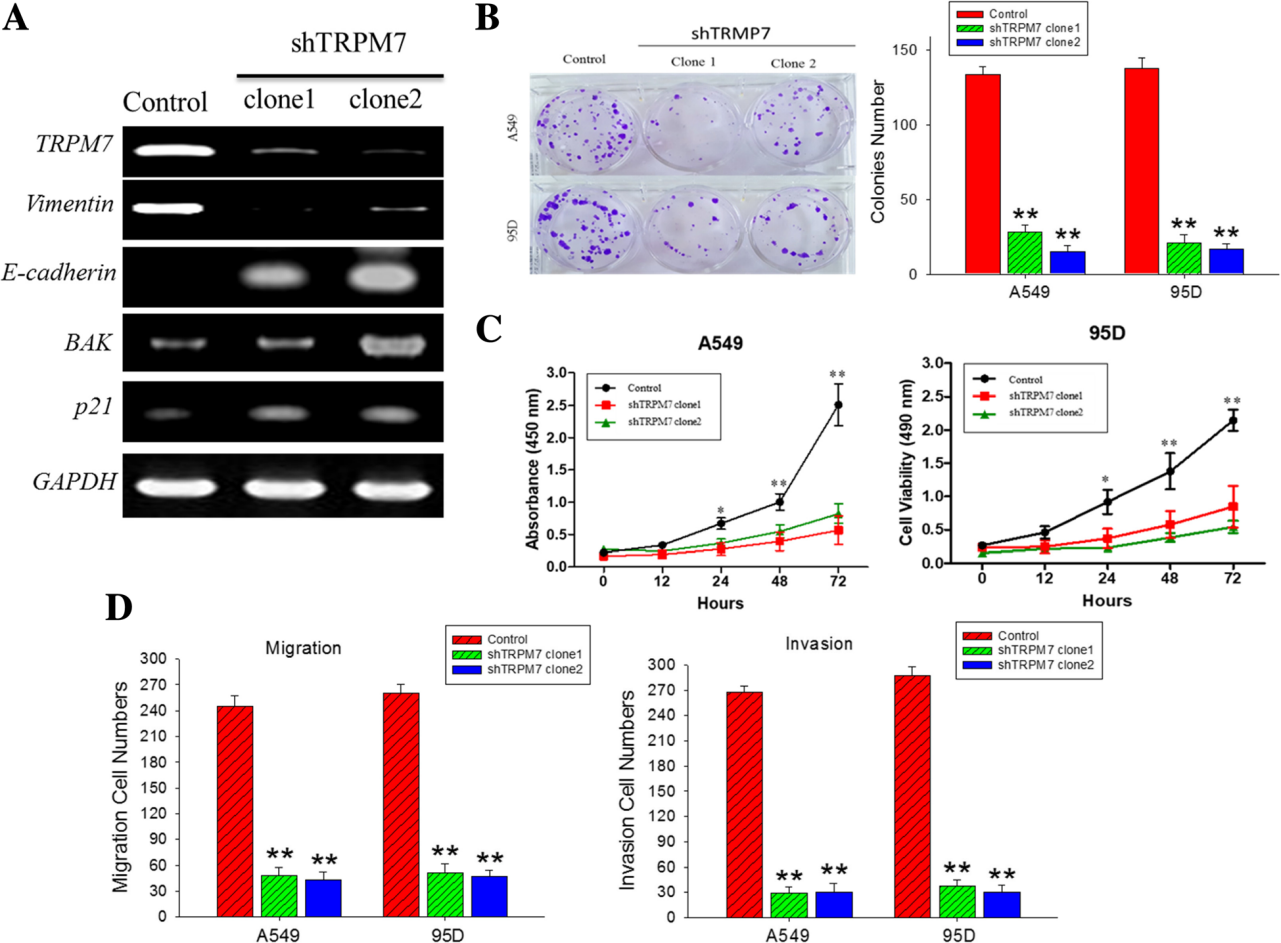
Downregulation of TRPM7 expression suppress the viability and metastatic phenotype of human lung cancer cells. **a** The effect of shTRPM7 clones 1 and 2 on the mRNA expression levels of *TRPM7, Vimentin, E-cadherin, p21* and *BAK.* **b** Fewer colonies were formed by the shTRPM7 clones compared to the control A549 and 95D cells. **c** Cell viability of A549 and 95D cells is significantly reduced in shTRPM7 clones, compared to the control cells. **d** shTRPM7 clones exhibited suppressed migration and invasion potential, in comparison to the control A549 and 95D cells. GAPDH served as loading control. * *p* < 0.05, ** *p* < 0.01, *** *p* < 0.001

In furtherance of our functional characterization of TRPM7 in lung cancer, we knocked down TRPM7 in A549 or 95D cells using the short hairpin RNA. Our results showed that downregulation of TRPM7 protein expression in the lung cancer cells, was associated with concomitant downregulation of *vimentin* mRNA expression, while the mRNA expression of *E-cadherin, p21* and *BAK* was upregulated (Fig. 3a). Since p21 is a key regulator of the cell cycle and associated with G1/G2 arrest [21] and BAK serves a pro-apoptotic function [22], rendering both as modulators of cell survival and proliferation, we thus assessed the effect of TRPM7 on the viability and proliferation of lung cancer cells using the SRB cell viability assay. We demonstrated that silencing TRPM7 in A549 or 95D cells significantly suppressed the ability of these cells to form colonies (*p* < 0.01) (Fig. 3b). We also demonstrated that compared to the control A549 cells, at the 72-h time-point, the shTRPM7 clones 1 and 2 exhibited 2.13-fold and 2.33-fold reduction in viability; similarly, for the 95D cells, the fold viability difference between control and shTRPM7 clones 1 and 2 was 1.80-fold and 2.00-fold (Fig. 3c). In addition, Silenced TRPM7 expression in lung cancer cells, markedly reduced their migration (~ 4.6-fold, *p* < 0.01) and invasion (~ 6.1-fold, p < 0.01) potential (Fig. 3d). This is corroborated by findings of our clinicopathological analysis showing that high TRPM7 expression positively correlates with lymph node metastasis (*p* = 0.005). These data are suggestive of the critical role of TRPM7 in the modulation of epithelial-to-mesnchymal transition (EMT), as well as the viability and metastatic phenotype of human lung cancer cells.

### TRPM7 regulates the CSCs activities of lung cancer cells by modulating the Hsp90α/uPA/MMP2 signaling pathway

**Fig. 4:**
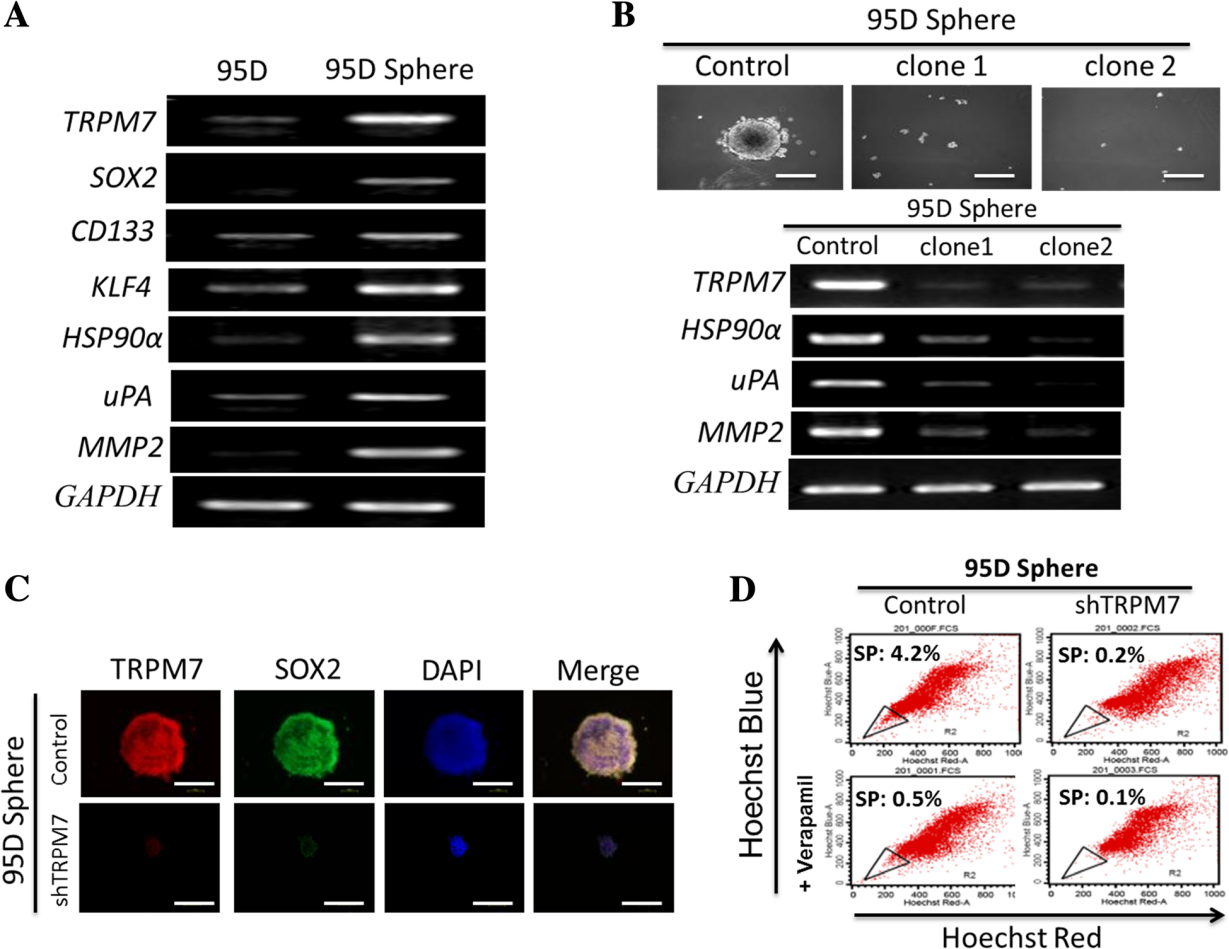
TRPM7 regulates the CSCs activities of lung cancer cells by modulating the Hsp90α/uPA/MMP2 signaling pathway. **a** Representative RT-PCR ananylsis showing upregulated *TRPM7, SOX2, CD133, KLF4, HSP90α, uPA*, and *MMP2* in 95D tumorspheres, compared to parental 95D cells. **b** Photo images showing shTRPM7 clones lost ability to form tumorspheres in comparison to the control 95D cells, *upper panel*; *TRPM7, HSP90α, uPA*, and *MMP2* mRNA expression is significantly downregulated in the tumorspheres derived from shTRPM7 clones, *lower panel*. **c** Immunoflorescence staining showing shTRPM7 95D tumorspheres were significantly smaller, compared to the control 95D tumorspheres. **d** Identification of side population (SP) cells from the control or shTRPM7 95D cells. SP cells were sorted by flow cytometry using Hoechst red and blue from 95D cells with or without verapamil pretreatment for 30 min. GAPDH served as loading control. * *p* < 0.05, ** *p* < 0.01, *** *p* < 0.001

Since lung cancer is a heterogeneous disease with the presence of CSCs being demonstrated in lung cancer and implicated in the metastatic and treatment-resistant phenotype of malignant lung cells (23), we examined if TRPM7 exhibits any modulatory effect on the CSCs- like phenotype of lung cancer cells and their associated aggressiveness, by evaluating the effect of TRPM7 on the protein expression of stemness markers. Results of our RT-PCR analysis demonstrate that the expression level of *TRPM7* mRNA was elevated upregulated in tumorspheres derived from 95D cells, compared to the control 95D cells, and this enhanced expression of *TRPM7* was associated with concomitant upregulation of *SOX2, CD133, KLF4,* heat-shock protein 90α *(HSP90α),* urokinase plasminogen activator *(uPA),* and matrix metalloproteinase 2 *(MMP2)* (Fig. 4a). In addition, we demonstrated that a correlation exists between TRPM7 expression, as TRPM7-expressing 95D cells readily formed tumorspheres, while the TRPM7 knockdown clones significantly lost their ability to form tumorspheres; furthermore, loss of tumorsphere formation ability was associated with significant reduction in *TRPM7, HSP90α, uPA*, and *MMP2* mRNA expression level (Fig. 4b). In similar experiment, using immunofluorescence staining, we showed that compared to the small tumorspheres formed by the shTRPM7 clones, tumorspheres derived from the control 95D cells were significantly larger in size, and were characterized by the nuclear co-localization of TRPM7 and SOX2, unlike the shTRPM7 tumorspheres (Fig. 4c). To further explore the effect of TRPM7 in the maintenance of CSCs-like lung SP cells, the human lung cancer cell line 95D was sorted by flow cytometry after incubation with Hoechst 33342 for 90 min. SP cells represented 4.2% of the total 95D control cells, while for the shTRPM7 clone, the SP cells were significantly reduced to only 0.2%. When preincubated with verapamil for 30 min, the proportion of SP cells was reduced to 0.5% of the total 95D control cells, or 0.1% for the shTRPM7 cells (Fig. 4d). These data suggest an association between the observed enhanced tumorsphere formation ability, increased expression of stemness markers, and upregulated TRPM7 expression, as well as indicate that TRPM7 regulates the CSCs activities of lung cancer cells by modulating the Hsp90α/uPA/MMP2 signaling pathway.

### TRPM7 inhibitor, Waixenicin a suppresses the CSCs phenotype of lung cancer cells

**Fig. 5:**
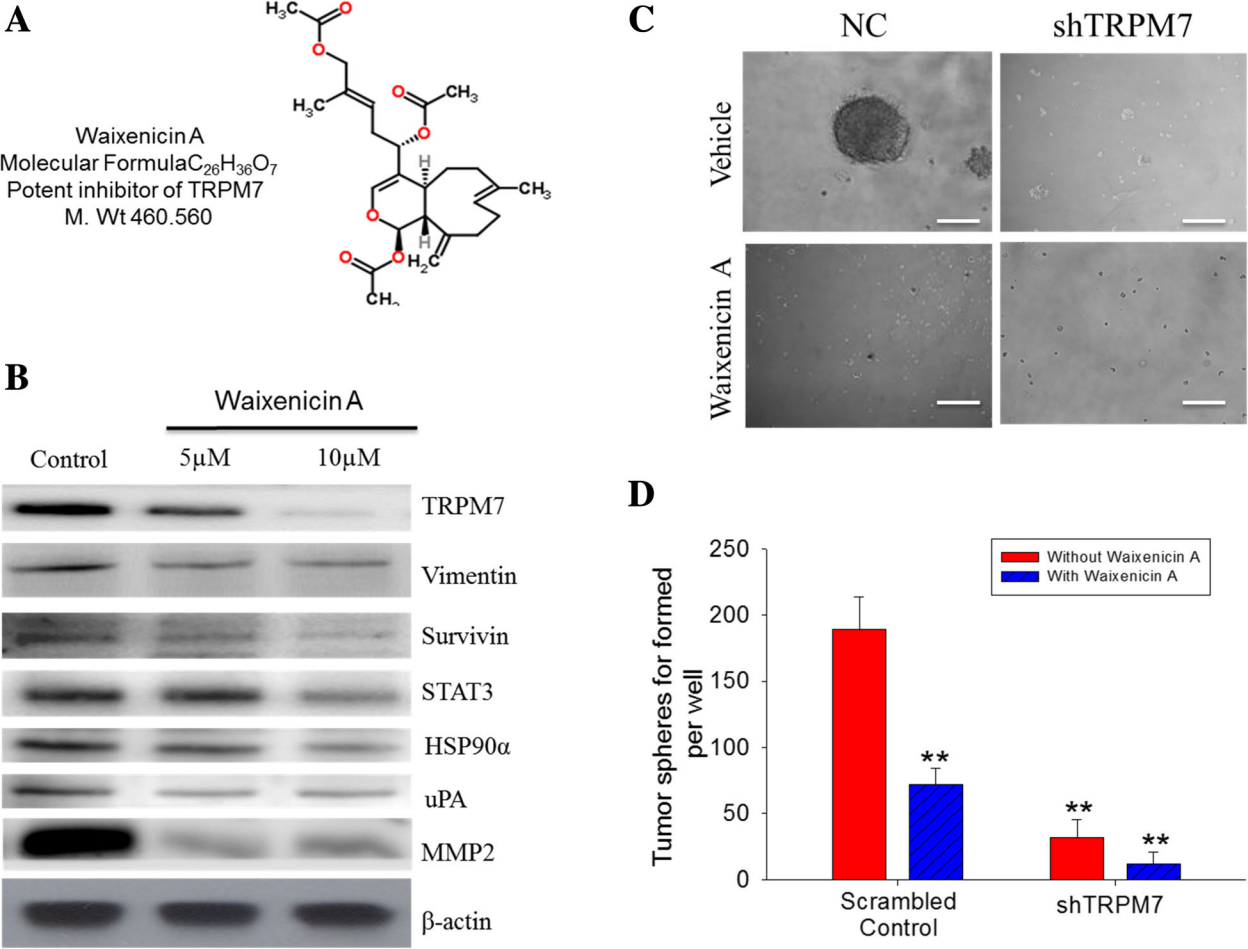
TRPM7 inhibitor, Waixenicin A suppresses the CSC phenotype of lung cancer cells. **a** Chemical structure of Waixenicin A with molecular formular C_26_H_36_O_7_ and molecular weight 460.56 mg/mol. **b** The inhibitory effect of 5 μM and 10 μM Waixenicin A on the expression levels of TRPM7, Vimentin, Survivin, STAT3, HSP90α, uPA, and MMP2 in 95D cells. **c** Photo image and (**d**) histography showing significantly inhibited tumorsphere formation by shTRPM7 and/or Waixenicin A treatment

Having shown that TRPM7 expression is relevant in the pathogenesis and prognosis of lung cancer, as well as inhibitory effects of TRPM7 knockdown in lung cancer cells, we then investigate the effect of TRPM7 inhibitor, Waixenicin A (Fig. 5a) on the TRPM7-rich 95D cells. Our results demonstrate that 48 h treatment of the 95D cells with 5 μM or 10 μM Waixenicin A significantly and dose-dependently inhibited the expression level of TRPM7, Vimentin, Survivin, STAT3, HSP90α, uPA, and MMP2 proteins, compared to the untreated control cells (Fig. 5b). In parallel experiments using the tumorsphere formation assay, we demonstrated that like the inhibitory effect of shTRPM7 on tumorsphere formation, Waixenicin A not only more efficiently suppressed tumorsphere formation by the lung cancer cells, but also enhanced the anti-CSCs effect of shTRPM7 (Fig. 5c and d). These results are indicative of the molecular and therapeutic targetability of TRPM7 in lung cancer, as well as further corroborate the anti-CSCs effect of targeting TRPM7 in lung cancer cells.

## Discussion

Despite the significant progress made in anticancer therapy, lung cancer ranking as the leading cause of cancer-related deaths in the United States, with a 5-year survival rate of 18.1% and a 25.9% death rate in 2017 (https://seer.cancer.gov/statfacts/html/lungb.html), remains a lethal medical challenge, especially as patients continue to be beleaguered with treatment failure, resistance to therapy and increased incidence of locally advanced and metastatic disease, which are not unrelated with the presence of a small population of lung cancer cells termed CSCs which are characterized by enhanced EMT/metastatic phenotype, facilitate tumor formation, growth and progression [23]. Thus, the needs for the discovery and development of therapeutic strategies that target these lung CSCs effectively.

In the present study, we provide evidence that, TRPM7 (i) is aberrantly expressed in lung cancer tissue samples and cell lines, (ii) is an independent indicator of poor prognosis in lung cancer, and (iii) regulates the CSCs activities of lung cancer cells by modulating the Hsp90α/uPA/MMP2 signaling pathway, while its molecular or therapeutic inhibition (iv) suppresses the viability as well as metastatic and CSCs phenotypes of human lung cancer cells, and (vi) enhances the anticancer effect of standard chemotherapeutics.

Ion channels continue to be implicated in cancer development and progression, being aberrantly expressed in tumor tissues or cell lines compared to normal tissues or cells, and are actively involved in cellular proliferation, evasion of cell death, metabolic reprogramming, neovascularization, and enhanced motility of tumor cells [12, 13]; interestingly, while the non-selective, cationic TRPM7 channel which mediates both Ca^2+^ and Mg^2+^ influx, has been shown to be overexpressed in several human malignancies, including breast, prostrate, pancreatic, and ovarian cancers, its expression profile and role in lung cancer development and progression remained unknown. In this study, we demonstrated that the preferential overexpression of TRPM7 in lung cancer tissues and cell lines is not only statistically significant but also clinically relevant, as it bears poor prognostic implications in patients with lung cancer (Figs. 1 and 2). This is consistent with data from our correlative clinicopathological analysis showing that TRPM7 positively correlated tumor grade, tumor size and lymph node metastasis; and is corroborated by the findings of Dhennin-Duthile et al. [13]., in which several TRPs including TRPM8 and TRPM7 were shown to be overexpressed in human breast cancer epithelial cells and tissues, with strong positive correlation with pathological parameters such as the Scarff-Bloom-Richardson (SBR) grade, Ki67 proliferative index and tumor size.

Furthermore, we showed that shRNA- mediated reduction of TRPM7 expression significantly suppressed the viability of lung cancer cells, attenuated the ability of the malignant cells to form colonies, and impeded their cell migration and invasion; all these were associated with concomitant downregulation of apoptotic marker Bak and mesenchymal markers Vimentin, and Snail, as well as upregulation of epithelial marker E-cadherin and tumor suppressor p21 (Fig. 3). Our finding is consistent with demonstrated upregulation of several transcription factors including Snail, Twist, Zeb, and Vimentin in metastatic cells undergoing EMT [24]. EMT itself entails the physiological or pathological conversion of an epithelial cell into an elongated cell with mesenchymal phenotype. Our data indicate that similar to TGF-β, TRPM7 plays a vital role in the activation of Snail, which in turn downregulates E-cadherin and Bak, while upregulating N-cadherin, p21 and Bcl-xL, and this process is involved in EMT. Similarly, our findings are corroborated by documented reversal of EMT status, with associated inhibition of cell proliferation, migration, and invasion, as well as enhanced cell cycle arrest at G0/G1 and apoptosis in human bladder cancer [25]. This TRPM7-associated expression profile is disease-relevant because the migration of cancer cells requires activation of genes necessary for differentiation, impede proliferative activities, induce anti-apoptotic mechanisms, transform the cells from epithelial to mesenchymal phenotype, suppress receptors that facilitate cell-to-cell attachment, degrade cell-to-cell junctions, and enhance cell motility [24].

Furthermore, we showed for the first time that TRPM7 regulates the CSCs activities of lung cancer cells by modulating the Hsp90α/uPA/MMP2 signaling pathway (Fig. 4a-d). Interestingly, among the genes that are regulated by *TRPM7, SOX2, CD133* and *KLF4* are cancer stemness markers. *HSP90α, uPA* and *MMP* mediates cell proinflammation, invasion, metastasis and drug resistance. The altered expression of these genes, as demonstrated in this study, may be reflective of the functional significance of TRPM7 in lung cancer cells, which to a large extent includes the maintenance of the lung cancer stem cell-like phenotypes and the suppression of lung metastasis. This is consistent with TRPM7’s documented induction role in the upregulation of CSCs markers such as CD133 and ALDH1, as well as promotes the proliferative, metastatic and CSCs-like phenotypes of GBM cells [17]. The uniqueness of TRPM7 lies in the fact that while it encodes an α-kinase domain fused to the ion channel moiety, the kinase and channel domain may be mutually regulated, however although the kinase domain contributes partially to the modulation of the channel sensitivity to Mg^2+^ and cAMP, it is not required for TRPM7 channel activity. Our results showing that TRPM7-mediated CSCs-like phenotype in lung cancer is modulated by Hsp90α/uPA/MMP2 signaling is therapeutically-relevant and partially consistent with recently demonstrated role of TRPM7 in the regulation of pancreatic cancer cell invasion through the Hsp90α/uPA/MMP2 pathway [26]. Hsp90 is a master regulator of cancer, and many Hsp90 target genes are nodes of oncogenic pathways [27]. By interacting with receptor tyrosine kinases, cytosolic Hsp90 plays vital roles in cell proliferation, differentiation, migration, and cancer progression. HSP90α is the secreted isoform and is associated with MMP2, implicating extracellular Hsp90 in cancer metastasis [28]. We speculate that the extracellular Hsp90 initiates EMT in lung cancer cells by modulating TRPM7 expression and activity. It is also possible that the surface Hsp90 interacts with the extracellular domain of TRPM7; thus, the disruption of this Hsp90/TRPM7 interaction results in the inhibition of cell invasion and associated with altered actin dynamics in the human lung cancer cells. In the context of TRPM7-mediated lung CSCs regulation as we have demonstrated, Hsp90 has been demonstrated to play an essential role in the regulation of pluripotency transcription factors, such as Oct4, Nanog, and Stat3 in mouse embryonic stem cells (ESCs) [29]. Similar to the findings in breast cancer stem cells [30], and consistent with its role in CSCs-derived primary tumor growth, we demonstrated that TRPM7-dependent targeting of highly expressed Hsp90α in lung CSCs inhibits tumorsphere formation, reduces the number of lung cancer SP cells and potentiates the anticancer effect of standard chemotherapeutics such as doxorubicin and cisplatin (Fig. 4a-d).

Recently, Zierler et al. [16] suggested that waixenicin A is a potent and relatively specific inhibitor of TRPM7 ion channels. In this recent study, waixenicin A was found to be a selective inhibitor of the TRPM7 ion channel, and patch-clamp experiments confirmed waixenicin A as a TRPM7 antagonist. However, the inhibitory effects of waixenicin A on TRPM7 are strongly dependent on intracellular Mg2+ concentration. Furthermore, it was also found that the pharmacological inhibition of TRPM7 by waixenicin A caused growth arrest in the G0/G1 phase (16). Our data confirm that Waixenicin A inhibits native TRPM7 currents in both lung cancer cells and its counterpart lung cancer spheres (Fig. 5a-d). These features make waixenicin A an attractive molecular structure for targeting TRPM7-related lung cancer stemness pathophysiology. This is clinically relevant since the most often lung cancer is a probable CSCs - rich and -driven pathology, therefore the preferential targeting and elimination of these CSCs which are implicated in resistance to standard anticancer therapy, metastasis and recurrence, by TRPM7 silencing or Waixenicin A treatment alone or in combination with conventional anticancer therapeutics, constitute a potential right step towards the development of a more efficient anti-cancer therapeutic strategy for treatment of advance staged, metastatic, or recurrent lung cancer.

## Conclusions

**Fig. 6:**
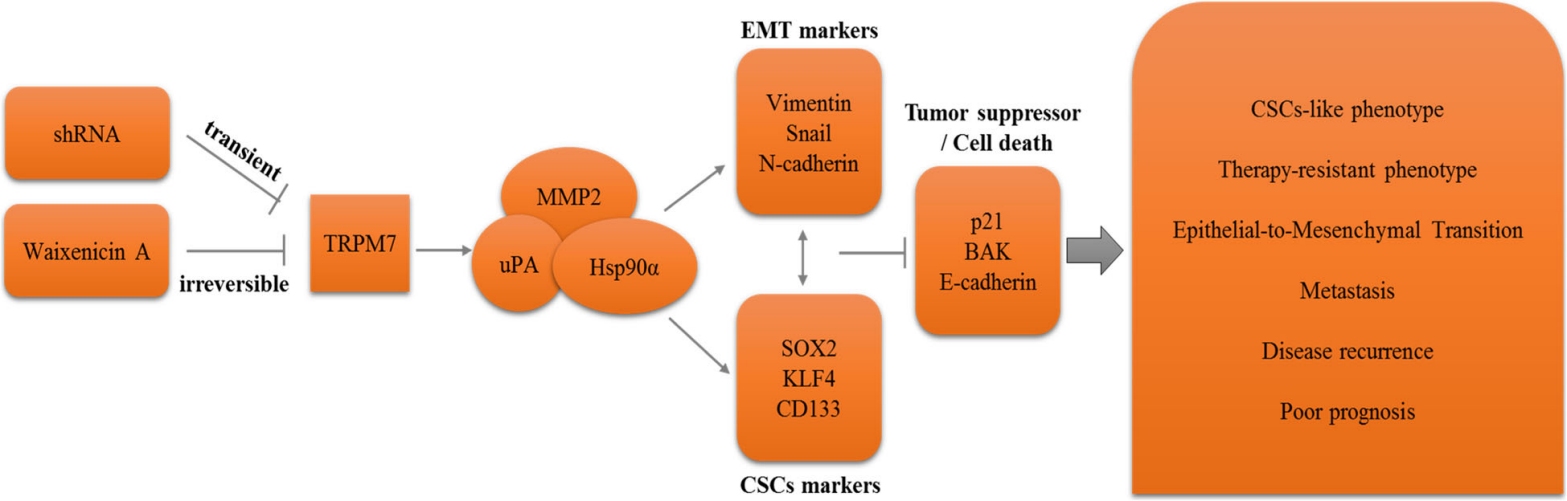
Pictorial Abstract showing that the molecular or therapeutic inhibition of TRPM7 expression and/or activity attenuates the cancer stem cell-like and metastatic phenotypes of lung cancer cells through the modulation of the Hsp90α/uPA/MMP2 signaling

In conclusion, as depicted in our schematic summary (Fig. 6), we have provided evidence that aberrant TRPM7 expression induces the cancer stem cell-like and metastatic phenotypes of aggressive human lung cancer cells through positive modulation of the Hsp90α/uPA/MMP2 signaling pathways and induction of pluripotency transcription factors. This results in decreased sensitivity of lung cancer cells to chemotherapy, enhanced formation of micro-metastases, disease recurrence and invariably poor prognosis. In essence, we showed that targeting TRPM7 inhibits oncogenic activity, disrupts EMT in favor of the benign epithelial phenotype, inhibit cancer metastasis, attenuate cancer stemness, and increase sensitivity to chemotherapy and potentially results in better prognosis for lung cancer patients.

## Additional file

Additional file 1: **Figure S1.** TRPM7 is an independent indicator of poor prognosis in lung cancer. (A) Kaplan-Meier analysis of TRPM7 gene expression in GSE37745 lung cancer dataset show patients with low TRPM7 expression had better overall survival than those with high TRPM7 expression. (B) Kaplan-Meier analysis of TRPM7 gene expression in GSE30219 lung cancer dataset show patients with low TRPM7 expression had longer relapse-free survival than those with high TRPM7 expression. (DOCX 184 kb)

## Abbreviations

CSCs: Cancer stem cells
EMT: Epithelial-to-mesnchymal transition
FITC: Luorescence isothiocyanate
GEO: Gene Expression Omnibus
HSP90α: **σ**Heat-shock protein 90α
MMP2: Matrix metalloproteinase 2
PVDF: Polyvinylidene difluoride
SDS-PAGE: Sodium dodecyl sulfate - polyacrylamide gel electrophoresis
TRP: Transient receptor potential
TRPM7: Transient receptor potential melastatin 7
uPA: Urokinase plasminogen activator
