# Supplementary figures and images for: TRPM7 overexpression enhances the cancer stem cell-like and metastatic phenotypes of lung cancer through modulation of the Hsp90α/uPA/MMP2 signaling pathway
Source: BMC Cancer. 2018 Nov 26;18:1167. doi: 10.1186/s12885-018-5050-x (PMC6258145; doi:10.1186/s12885-018-5050-x)

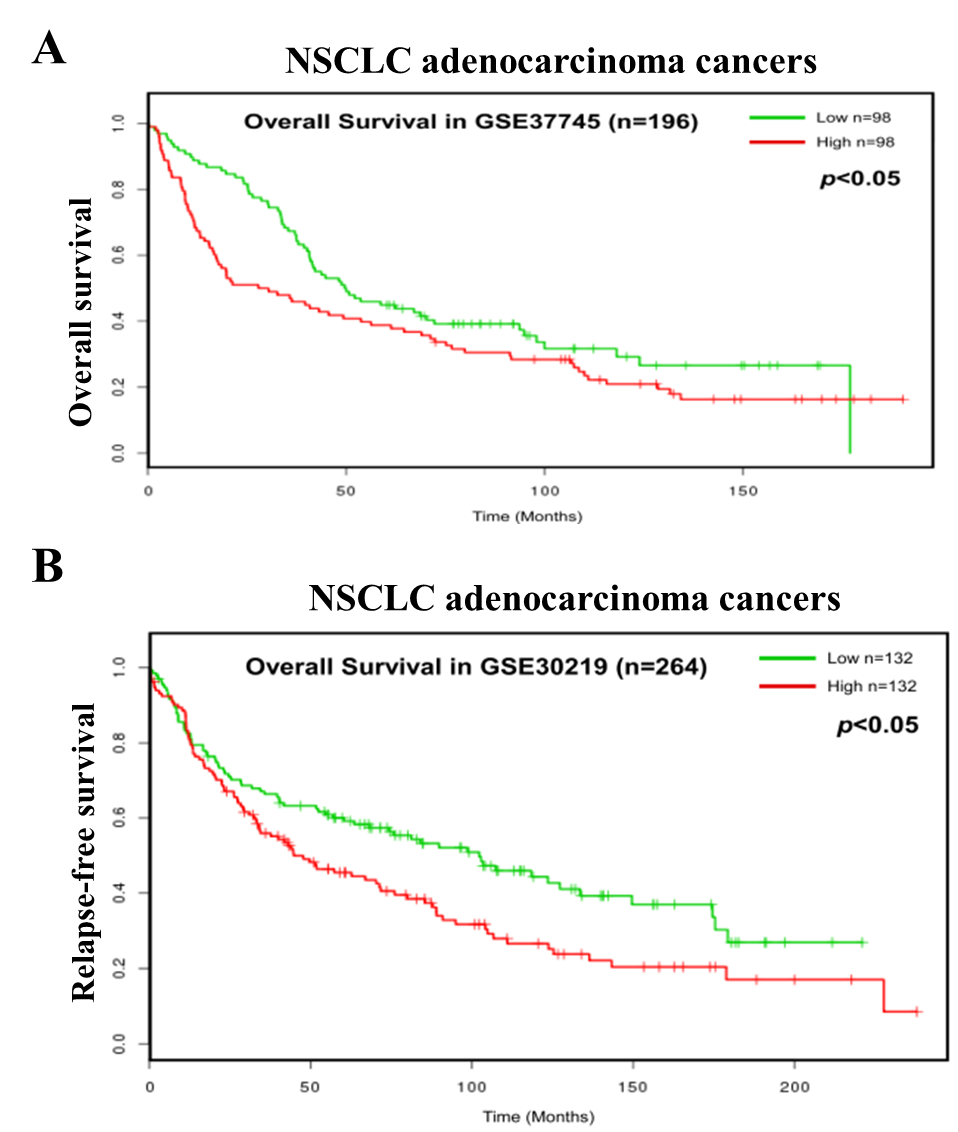


**Supplementary Figure S1**

Supplement: Supplementary file 1 — Figure S1. TRPM7 is an independent indicator of poor prognosis in lung cancer. (A) Kaplan-Meier analysis of TRPM7 gene expression in GSE37745 lung cancer dataset show patients with low TRPM7 expression had better overall survival than those with high TRPM7 expression. (B) Kaplan-Meier analysis of TRPM7 gene expression in GSE30219 lung cancer dataset show patients with low TRPM7 expression had longer relapse-free survival than those with high TRPM7 expression. (DOCX 184 kb) [file 12885_2018_5050_MOESM1_ESM.docx]
